# MPO deficiency confers impaired processing of neutrophil reactive oxygen species in a patient with severe CRMO

**DOI:** 10.1186/1546-0096-13-S1-P191

**Published:** 2015-09-28

**Authors:** S Berg, H Björnsdottir, M Sundqvist, P Wekell, K Christenson, V Osla, A Welin, J Bylund, A Karlsson

**Affiliations:** 1 University of Gothenburg, Dept Pediatrics, Gothenburg, Sweden; 2 University of Gothenburg, Dept Rheumatology and Inflammation Research, Gothenburg, Sweden; 3 NU-Hospital Organization, Dept Pediatrics, Uddevalla, Sweden; 4 University of Gothenburg, Dept Oral Microbiology and Immunology, Gothenburg, Sweden

## Introduction

We report a severe case of chronic recurrent multifocal osteomyelitis (CRMO) associated with total myeloperoxidase (MPO) deficiency. The etiology of CRMO is in most cases unknown, and this is to our knowledge the first case associated with MPO-deficiency. Leukocyte MPO-deficiency renders neutrophils unable to process superoxide to secondary reactive oxygen species (ROS). Partial MPO deficiency is seldom associated with pathology but little is known about the effects of total MPO deficiency.

## Objectives

To increase our understanding of disease mechanisms in CRMO by describing symptoms, treatment outcomes, basic inflammatory parameters, and innate immune cell function in this patient.

## Patient and methods

The patient, a girl of 18 years, was healthy until the age of 10 when she developed bilateral swollen and painful thighs. After a four-year period with numerous clinical investigations she was diagnosed with CRMO. At the time, she suffered from severe weight loss (BMI 10.5) and showed elevated inflammatory markers (ESR 80 mm/h and CRP 54 mg/L). Radiology showed bilateral inflammation of femur and biopsy showed unspecific inflammation. The patient displayed complete MPO deficiency (genetically confirmed).

Blood counts, inflammatory markers and neutrophil function were assessed by standard laboratory techniques. Samples were analyzed both during a flare, induced by withdrawal of TNF-blockade, and after treatment reintroduction.

## Results

The patient responded extremely well to TNF-blockade, but not to IL-1 blockade, both clinically and in terms of inflammatory markers. The treatment did however not abrogate the underlying inflammatory trigger as symptoms returned after treatment withdrawal.

Comparing neutrophil function during a flare and under treatment, no substantial differences was detected, neither in primary ROS production, nor in surface marker exposure. The inability to process ROS due to MPO deficiency resulted in impaired formation of neutrophil extracellular traps (NETs), is suggested to be of importance for bacterial clearance.

## Conclusion

Our clinical data indicate that inflammation was driven by TNFa rather than IL-1 in the patient. The laboratory results confirmed the inability of MPO-deficient neutrophils to form NETs, but further studies are needed to elucidate the possible causal relationship between CRMO and MPO/NET formation.

